# Cultivar-Dependent Variation in Phenolic Compounds, Anthocyanin Profile, and Fruit Quality Traits in Romanian Blueberry (*Vaccinium corymbosum* L.)

**DOI:** 10.3390/molecules31122068

**Published:** 2026-06-12

**Authors:** Oana-Crina Bujor, Mihaela Iordăchescu, Andrei Cătălin Petre, Anca Amalia Udriște, Adrian Asănică, Liliana Bădulescu

**Affiliations:** 1 Research Center for Studies of Food and Agricultural Products Quality, University of Agronomic Sciences and Veterinary Medicine of Bucharest, 59, Mărăști Blvd., 011464 Bucharest, Romania; oana.bujor@qlab.usamv.ro (O.-C.B.); mihaela.iordachescu@qlab.usamv.ro (M.I.); andrei.petre@qlab.usamv.ro (A.C.P.); amalia.udriste@qlab.usamv.ro (A.A.U.); 2 Faculty of Horticulture, University of Agronomic Sciences and Veterinary Medicine of Bucharest, 59, Mărăști Blvd., 011464 Bucharest, Romania; adrian.asanica@horticultura-bucuresti.ro

**Keywords:** *Vaccinium corymbosum* L., fruit quality, polyphenols, anthocyanins, Folin–Ciocalteu, DPPH antiradical capacity, UPLC-PDA, genotypic variation

## Abstract

The blueberry (*Vaccinium corymbosum* L.) is known for its high content of bioactive compounds, which are widely recognized for their health-promoting properties. This study aimed to characterize the fruit quality, total phenolic content (TPC), total monomeric anthocyanin content (TMA), anthocyanin profile and antioxidant activity of the nine Romanian *V. corymbosum* genotypes (‘Augusta’, ‘Azur’, ‘Delicia’, ‘Lax’, ‘Pastel’, ‘Prod’, ‘Safir’, ‘Simultan’, and ‘Vital’) over three consecutive harvest seasons (2023–2025). Significant genotype- and year-dependent variation was observed for all parameters. ‘Lax’ consistently accumulated the highest total anthocyanin content across all three seasons, while ‘Simultan’ exhibited the highest antioxidant activity and total monomeric anthocyanin content. ‘Prod’ consistently recorded the lowest phytochemical values despite achieving the highest firmness in 2025. UPLC analysis identified 10 anthocyanins, covering all five major anthocyanidin classes. Strong positive correlations were found between TPC, TMA, and antioxidant activity. These results confirm that genotype is the primary determinant of blueberry phytochemical composition, as indicated by the largest effect sizes in the two-way ANOVA, with harvest year and genotype × year interaction as statistically significant but secondary modulating factors, and identify ‘Lax’, ‘Simultan’, and ‘Safir’ as promising cultivars for nutraceutical and breeding applications.

## 1. Introduction

The blueberry (*Vaccinium corymbosum* L.) is among the most economically important small fruit crops worldwide, valued not only for its sensory qualities but also for its nutritional and functional profile. The fruit is recognized as a rich dietary source of polyphenols, including anthocyanins, flavonols, hydroxycinnamic acid derivatives, and proanthocyanidins, as well as organic acids, sugars, vitamins, and minerals [1,2]. Regular consumption of *Vaccinium* fruits is associated with a lower risk of cardiovascular disease, type 2 diabetes, obesity, and neurodegenerative disorders [3,4]. This accumulating evidence has contributed to increased consumer demand and has markedly expanded the global cultivation area of highbush blueberries over the past two decades. Epidemiological and clinical studies indicate that consuming moderate amounts of blueberries regularly leads to improved vascular function, glycaemia regulation, and neuroprotection [3]. The protective mechanisms of *V. corymbosum* polyphenols include inhibition of endothelial inflammation, reduction in oxidative damage, and improvement of lipid profiles [5].

In Romania, blueberry cultivation has expanded considerably in recent years, driven by favourable climatic and pedological conditions and an increasing market demand for fresh fruit consumption and a growing export market [6,7,8]. Systematic blueberry breeding has been carried out at the Research Institute for Fruit Growing Pitești-Mărăcineni since 1980, resulting in a national collection of cultivars obtained through controlled hybridization: ‘Lax’, ‘Prod’, ‘Vital’, ‘Azur’, ‘Simultan’, ‘Delicia’, ‘Pastel’, ‘Safir’, and ‘Augusta’ [9]. Although the cultivars examined are a nationally registered Romanian set, they share genetic lineages with widely used American parents, and their phytochemical characterization under Romanian pedoclimatic conditions contributes new data to the global literature on genotype × environment interactions in *V. corymbosum*. Moreover, the integrative approach combining molecular genomic data with phenolic profiling and fruit quality traits provides a methodological framework applicable to any regional cultivar collection. While the agronomic performance of these cultivars has been documented, their molecular genetic characterization has only recently been initiated. Whole-genome resequencing of seven Romanian genotypes identified multiple differences in genes involved in fruit growth and development concentrated in a hotspot of genomic variation on scaffold 22 [10]. Similar to studies for *Vaccinium* sp. molecular characterization that were performed elsewhere, such as genetic diversity studies using molecular markers [11,12], ISSR marker analysis confirmed substantial genetic variability across the Romanian cultivar set [13], and a subsequent SRAP marker study of all nine cultivars revealed polymorphism levels between 54% and 80%, grouping them into two main clusters with ‘Pastel’ as the most genetically distinct genotype [14]. Despite this growing genomic knowledge base, the relationship between the genetic background of these cultivars and their fruit quality or phytochemical profiles has not yet been investigated.

The phytochemical composition of *Vaccinium* fruits is shaped by a combination of genetic, environmental, and agronomic factors. Genetic constitution determines the cultivar-specific biosynthetic capacity for individual phenolic classes, whereas environmental conditions, including altitude, light exposure, temperature, and management practices, modulate their actual accumulation in harvested fruit [15,16]. For *V. corymbosum* specifically, it was demonstrated that genotype is the primary driver of antioxidant capacity and the content of anthocyanins, flavonols, and hydroxycinnamic acid derivatives, while cultivation system exerts additional cultivar-specific effects [13]. This genotype × environment interaction was further confirmed by Ochmian et al. [17], who profiled 37 phenolic compounds across 19 highbush blueberry genotypes under harmonised orchard conditions and found that origin and cultivar jointly determined the polyphenolic chemotype and antioxidant capacity. In closely related wild *Vaccinium* species collected from Romanian mountain region, the structural diversity and quantitative distribution of phenolic compounds was documented across different morphological parts and harvest periods. In bilberries (*Vaccinium myrtillus* L.), anthocyanins dominate the fruit phenolic pool, while caffeic acid derivatives prevail in leaves and flavanol oligomers account for more than half of the phenolic compounds in stems, with seasonal effects particularly pronounced in leaves [18,19]. Another study on lingonberries (*Vaccinium vitis-idaea* L.) highlighted arbutin as the predominant leaf constituent and flavanols as the major stem and fruit compounds, and revealed only weak seasonal variations across two consecutive years [20]. Taken together, these findings underscore both the species-specific character of *Vaccinium* phenolic profiles and the central importance of genetic background in determining phytochemical composition [21,22,23].

Beyond phenolic composition and antioxidant activity, fruit quality encompasses a broader set of physicochemical attributes such as fruit weight, diameter, firmness, soluble solids content, titratable acidity, and colour parameters, that are decisive for both fresh-market acceptability and processing suitability. These traits are highly cultivar-dependent and interact directly with the phytochemical profile, since anthocyanin pigmentation governs fruit colour and the soluble solids–acidity balance determines flavour perception. Because these traits are quantitatively determined and highly responsive to environmental conditions, their phenotypic expression reflects a complex interaction between genetic background and external factors. Redpath et al. [24] demonstrated that genotype was a significant source of variation for most fruit quality characteristics and that year, harvest, and their interactions significantly affected berry weight, firmness, SSC, acidity, and color, emphasizing that annual climatic variation plays a major role in shaping fruit phenotype. Similarly, Ordóñez-Díaz et al. [25] reported that blueberry quality characteristics such as firmness, soluble solids, and acidity varied across seasons and cultivation systems, confirming the sensitivity of these parameters to environmental conditions. Cardeñosa et al. [26] further showed that genotype is a principal determinant of phenolic composition and antioxidant capacity, although cultivation system and irrigation regime can substantially modulate these biochemical responses. In addition, Cosmulescu et al. [7] observed significant cultivar × year effects on blueberry production characteristics under Romanian conditions, highlighting the importance of evaluating cultivar performance over multiple seasons to capture annual variability. Therefore, annual variations in blueberry fruit quality are significant and are often driven by the interaction between genotype, environment, and yearly climatic fluctuations. Comprehensive simultaneous assessments combining quality parameters with detailed polyphenolic profiling have been conducted for established international cultivar sets. Lachowicz-Wiśniewska et al. [27] profiled 75 bioactive compounds including 15 anthocyanins, 14 flavonols, phenolic acids, organic acids, and sugars in 14 northern highbush cultivars using UPLC-PDA-ESI-MS/MS, demonstrating that phytochemical composition and biological activities depended strongly on cultivar. Ochmian et al. [17] integrated phenolic subclass profiling with firmness, skin puncture resistance, soluble solids, and CIE colour parameters to illustrate how fruit quality and polyphenolic composition co-vary across genotypes.

For Romanian *V. corymbosum* material specifically, the only published biochemical characterisation of fruit to date concerns F1 hybrids derived from a single ‘Simultan’ × ‘Duke’ cross, for which Hera et al. [28] reported fruit weight, soluble solids, pH, vitamin C, total polyphenols, total flavonoids, total anthocyanins, and antioxidant activity. More recently, intraspecific hybridisation between additional Romanian cultivars and exogenous parents has provided preliminary heritability data for some of these traits [29]. Investigating these relationships is crucial for identifying cultivars that combine superior fruit quality with phenotypic stability across contrasting seasons, thereby providing a robust foundation for breeding programs, cultivar recommendation, and the optimization of management practices aimed at maximizing both market quality and functional value. However, a simultaneous, comprehensive characterisation of both fruit quality parameters and individual anthocyanins profiles across the full set of registered Romanian *V. corymbosum* cultivars grown under the same conditions has not yet been carried out.

In this context, the present study aimed to assess physico-chemical quality parameters of ripe fruits from the nine registered Romanian *V. corymbosum* genotypes grown under the same conditions, determine the content and composition of anthocyanins using UPLC-PDA, evaluate antioxidant activity by the DPPH radical-scavenging test and the Folin–Ciocalteu method for total phenolic content and examine the relationships between cultivar genotype, fruit quality traits, and phytochemical profile.

## 2. Results

### 2.1. Variation in Fruit Physical Characteristics in Blueberry Genotypes

**Figure 1:**
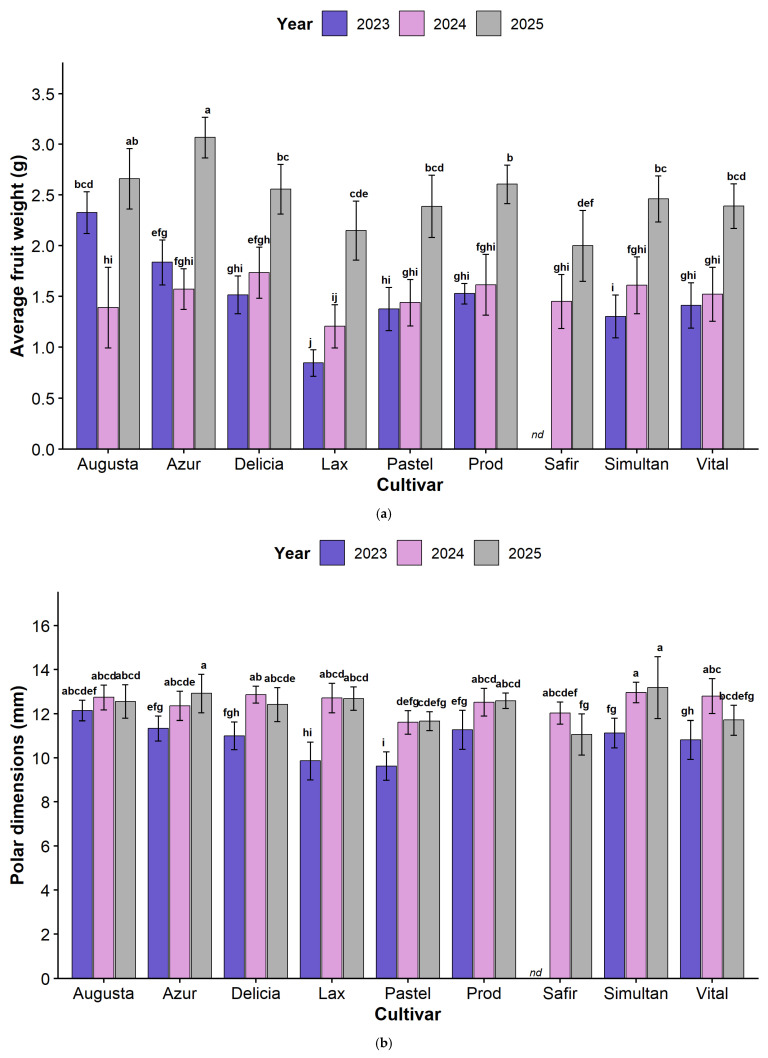

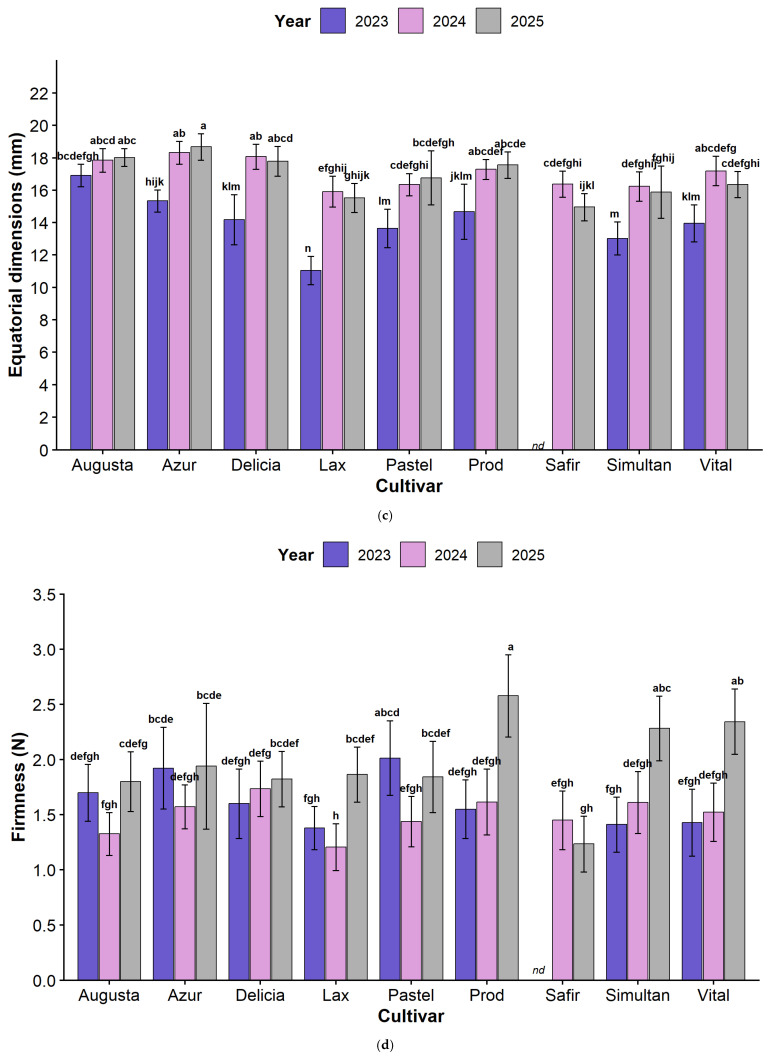
Influence of the blueberry genotypes on blueberry physical parameters (means ± SD, *n* = 7–10). (**a**) Average fruit weight; (**b**) Polar (height, h) dimensions; (**c**) Equatorial (transversal diameter, D) dimensions; (**d**) Firmness. Different letters indicate significant differences at *p* < 0.05 according to two-way ANOVA followed by Tukey’s HSD test. n.d.: not determined.

Fruit weight differed significantly among the nine *V. corymbosum* genotypes and across harvest years (Figure 1a). A two-way ANOVA revealed highly significant effects of genotype, harvest year, and their interaction together accounting for 84.4% of the total variance in fruit mass (R^2^ = 0.844, Table A1). In 2023, ‘Augusta’ produced the heaviest fruits (2.33 g), while ‘Lax’ recorded the lowest values (0.85 g). In 2024, fruit weight did not differ significantly from 2023 for most genotypes, with the exception of ‘Augusta’, which showed a significant decrease (from 2.33 g to 1.39 g), while values for the remaining cultivars ranged from 1.21 g (‘Lax’) to 1.74 g (‘Delicia’). In 2025, a significant increase in fruit weight was observed across all genotypes relative to 2023 and 2024, with ‘Azur’ recording the highest value (3.07 g), significantly exceeding most other genotypes. Values for the remaining cultivars ranged from 2.15 g (‘Lax’) to 2.66 g (‘Augusta’), without significant differences among several of them. Polar dimensions differed significantly among genotypes and across harvest years (Figure 1b). A two-way ANOVA revealed highly significant effects of genotype, harvest year, and their interaction, together accounting for 66.0% of the total variance in polar dimensions (R^2^ = 0.660, Table A1). In 2023, values ranged from 9.63 mm (‘Pastel’) to 12.15 mm (‘Augusta’). Several genotypes showed significantly lower polar dimensions in 2023 compared to subsequent years, most notably ‘Pastel’ (9.63 mm), ‘Lax’ (9.86 mm), and ‘Delicia’ (11.00 mm), which increased significantly in 2024 and 2025. By contrast, ‘Augusta’ showed no significant inter-annual variation. In 2025, ‘Simultan’ (13.18 mm) and ‘Azur’ (12.92 mm) recorded the highest polar dimensions, without significant differences between them, while values for the remaining cultivars ranged from 11.06 mm (‘Safir’) to 12.69 mm (‘Lax’), without significant differences among several of them. Equatorial dimensions differed significantly among genotypes and across harvest years (Figure 1c). A two-way ANOVA revealed highly significant effects of genotype, harvest year, and their interaction, together accounting for 78.1% of the total variance (R^2^ = 0.781, Table A1). In 2023, values ranged from 11.04 mm (‘Lax’) to 17.02 mm (‘Augusta’). Most genotypes showed significantly lower equatorial dimensions in 2023 compared to 2024 and 2025, except Augusta. There were no significant differences between 2024 and 2025 for any of the genotypes. Fruit firmness differed significantly among genotypes and across harvest years (Figure 1d). A two-way ANOVA revealed highly significant effects of genotype, harvest year, and their interaction, together accounting for 56.6% of the total variance (R^2^ = 0.566, Table A1). For all genotypes except for ‘Pastel’, which showed a significant decrease in 2024 relative to 2023, firmness values did not differ significantly between 2023 and 2024. In 2025, ‘Prod’ (2.58 N), ‘Simultan’ (2.30 N) and ‘Vital’ (2.35 N) showed a significant increase in firmness relative to previous years.

### 2.2. Variation in Fruit Chemical Characteristics in Blueberry Genotypes

Dry matter content showed significant variation among genotypes and harvest years (Table A1). For most genotypes, DM values did not differ significantly across years. ‘Lax’ was the only exception, showing significantly higher DM in 2023 (19.98%) compared to 2024 (14.26%) and 2025 (14.19%) (Table A2).

**Figure 2:**
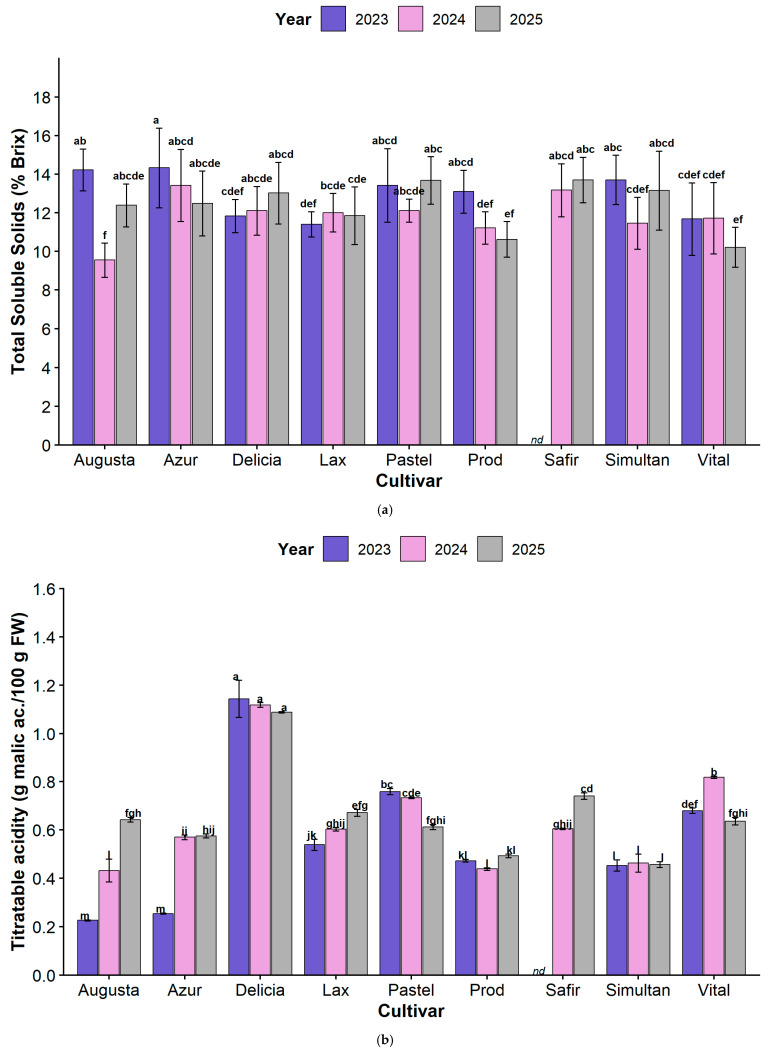
Influence of the blueberry genotypes on blueberry TSS and TA (means ± SD, *n* = 3–10). (**a**) Total Soluble Solids; (**b**) Titratable Acidity. Different letters indicate significant differences at *p* < 0.05 according to two-way ANOVA followed by Tukey’s HSD test. n.d.: not determined.

Total soluble solids (TSS) differed significantly among genotypes and across harvest years (Figure 2a). A two-way ANOVA revealed highly significant effects of genotype, harvest year, and their interaction, together accounting for 47.6% of the total variance (R^2^ = 0.476, Table A1). In 2023, TSS values ranged from 11.41% Brix (‘Lax’) to 14.34% Brix (‘Azur’). For most genotypes, TSS did not differ significantly across years. The most notable exception was ‘Augusta’, which showed a significant decrease in 2024 (9.55% Brix), representing the lowest value recorded across all genotypes and years. ‘Prod’ also showed a significant decrease in 2025 relative to 2023 (10.63% Brix). Titratable acidity (TA) showed the greatest inter-genotype variation among all chemical parameters measured (Figure 2b). A two-way ANOVA revealed highly significant effects of genotype, harvest year, and their interaction, together accounting for 99.4% of the total variance (R^2^ = 0.994, Table A1). ‘Delicia’ was consistently the most acidic cultivar across all three years (1.14, 1.12, and 1.09 g malic ac./100 g FW in 2023, 2024, and 2025, respectively), without significant inter-annual variation. ‘Augusta’ recorded the lowest acidity in 2023 (0.23 g/100 g FW), increasing significantly in 2024 (0.43 g/100 g FW) and again in 2025 (0.64 g/100 g FW), with significant differences among all three years. ‘Azur’ also recorded low acidity in 2023 (0.26 g/100 g FW), increasing significantly in 2024 and 2025, without significant differences between these two years. ‘Vital’ showed a significant increase in 2024 (0.82 g/100 g FW) relative to 2023 and 2025. ‘Prod’ and ‘Simultan’ showed no significant inter-annual variation. ‘Safir’ showed a significant increase from 2024 (0.60 g/100 g FW) to 2025 (0.74 g/100 g FW).

### 2.3. Variation in Phenolic Content, Anthocyanins, and Antioxidant Activity in Blueberry Fruit Genotypes over Three Harvest Years

Total phenolic content (TPC), total monomeric anthocyanin content (TMA), antioxidant activity (AA), and the sum of individual anthocyanin compounds were assessed in nine blueberry genotypes (Augusta, Azur, Delicia, Lax, Pastel, Prod, Safir, Simultan, and Vital) over three consecutive harvest seasons (2023, 2024, and 2025). Significant variation was observed among blueberry genotypes and harvest years for all analyzed parameters. Data for TPC, TMA, and AA are available only for the 2024 and 2025 seasons. The sum of individual anthocyanin compounds, quantified by UPLC-PDA, was assessed across all three years. All results are presented in Table 1.

#### 2.3.1. Genotypic Variation in TPC, TMA, and Antioxidant Activity over Harvest Years

The TPC was determined in all nine genotypes during the 2024 and 2025 harvest seasons (Table 1). In 2024, TPC values varied significantly among cultivars, ranging from a minimum of 200.17 mg GAE/100 g FW in ‘Prod’ to a maximum of 353.06 mg GAE/100 g FW in ‘Safir’ (Table 1). In 2025, ‘Augusta’ recorded the highest TPC (441.19 mg GAE/100 g FW), while ‘Prod’ showed the lowest value (183.82 mg GAE/100 g FW). Statistically significant differences among genotypes were detected within each harvest year, as indicated by distinct letter groupings in Table 1. In 2024, ‘Prod’ was assigned to the lowest statistical group (C), shared with ‘Augusta’ and ‘Azur’, indicating no significant difference among these three genotypes. In 2025, ‘Prod’ recorded the lowest TPC value and was assigned to a separate statistical group (d), significantly differing from all other genotypes.

The TMA was also determined for the 2024 and 2025 seasons (Table 1). In 2024, TMA ranged from 98.24 mg CGE/100 g FW in ‘Prod’ to 292.39 mg CGE/100 g FW in ‘Simultan’, with statistically significant differences detected among genotypes (Table 1). In 2025, ‘Lax’ recorded the highest TMA (312.77 mg CGE/100 g FW), while ‘Prod’ showed the lowest value (103.81 mg CGE/100 g FW). Notably, ‘Lax’ recorded the highest TMA values in both harvest years, whereas ‘Prod’ consistently ranked lowest among all genotypes.

Antioxidant activity (AA) determined by the DPPH test was measured in the 2024 and 2025 seasons (Table 1). In 2024, AA ranged from 2736.98 mg TE/100 g FW in ‘Prod’ to 4709.42 mg TE/100 g FW in ‘Simultan’, with statistically significant differences detected among genotypes (Table 1). In 2025, ‘Simultan’ recorded the highest AA (4470.53 mg TE/100 g FW), followed by ‘Safir’ (4312.07 mg TE/100 g FW), while ‘Prod’ consistently showed the lowest value (2698.56 mg TE/100 g FW). Across both harvest years, ‘Simultan’ and ‘Safir’ consistently ranked among the highest, whereas ‘Prod’ ranked lowest among all genotypes. Statistical analysis revealed significant differences among several genotypes in both years, as indicated by distinct and overlapping letter groupings in Table 1. In 2024, ‘Simultan’ and ‘Prod’ were assigned to non-overlapping statistical groups (A and D, respectively), representing the extremes of antioxidant activity, while the remaining genotypes occupied intermediate and partially overlapping groups. In 2025, a similar pattern was observed, with ‘Simultan’ and ‘Prod’ again assigned to non-overlapping groups (a and e, respectively).

**Figure 3:**
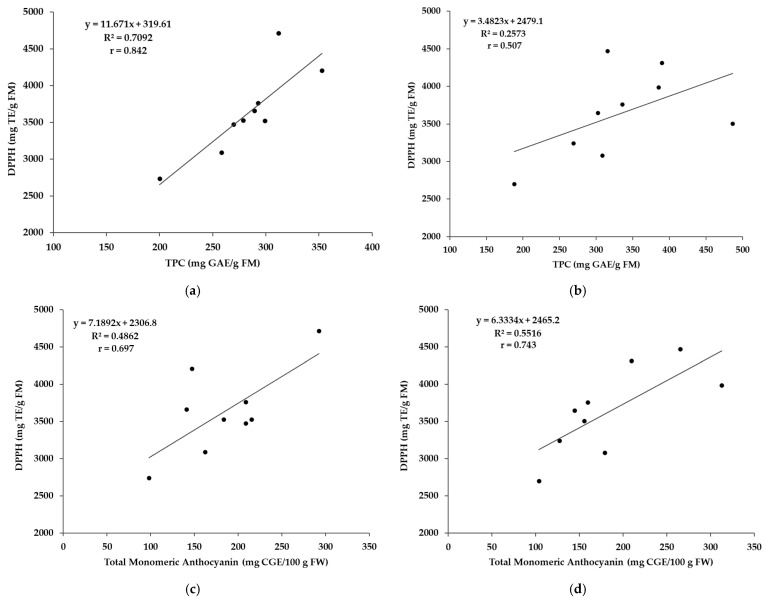
Correlation between total phenolic content (TPC) and antioxidant activity (DPPH) in *V. corymbosum* genotypes in 2024 (**a**) and 2025 (**b**), and between total monomeric anthocyanin content (TMA, pH differential method) and antioxidant activity (DPPH) in 2024 (**c**) and 2025 (**d**). Dashed line: linear regression. Pearson’s r and R^2^ indicated per panel.

Pearson correlation analysis revealed a strong and moderate positive relationship between TPC and antioxidant activity in the nine genotypes (Figure 3a,b), with r = 0.842 (R^2^ = 0.709) in 2024 and r = 0.507 (R^2^ = 0.257) in 2025, confirming the dominant contribution of phenolic compounds to DPPH radical-scavenging capacity in the studied blueberries. A similarly strong positive correlation was found between TMA and antioxidant activity (r = 0.697, R^2^ = 0.486 in 2024; r = 0.743, R^2^ = 0.552 in 2025; Figure 3c,d), consistent with the established role of anthocyanins as primary contributors to antioxidant activity in *V. corymbosum* [27,30,31].

#### 2.3.2. Genotypic Variation in Anthocyanin Profile and Content by UPLC-PDA over Harvest Years

**Figure A1:**
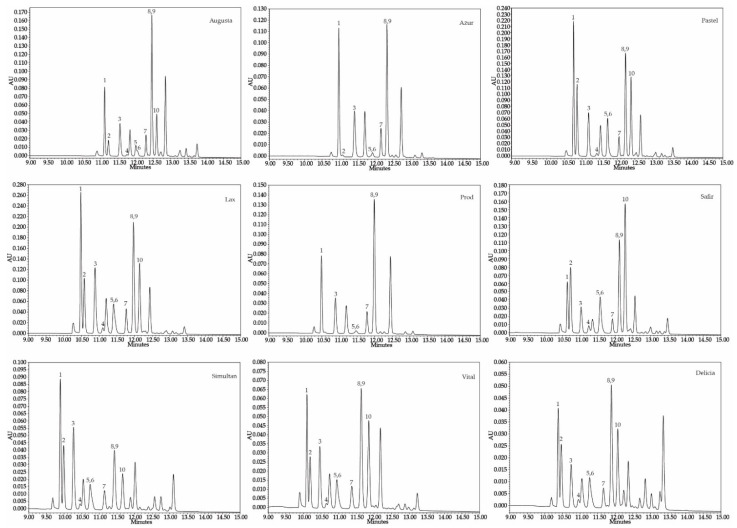
Chromatographic profile of anthocyanins in blueberry fruit genotypes, at 520 nm. Peak identification based on anthocyanin standards: 1—delphinidin-3-*O*-galactoside; 2—delphinidin-3-*O*-glucoside; 3—cyanidin-3-*O*-galactoside; 4—cyanidin-3-*O*-glucoside; 5—petunidin-3-*O*-glucoside; 6—cyanidin-3-*O*-arabinoside; 7—peonidin-3-*O*-galactoside; 8—peonidin-3-*O*-glucoside; 9—malvidin-3-*O*-galactoside; 10—malvidin-3-*O*-glucoside.

The qualitative anthocyanin profile of the nine blueberry fruit genotypes was assessed by UPLC-PDA analysis (Figure A1). In all fruits of blueberry 17 anthocyanins were tentatively identified by comparison of their UV-visible spectra and the literature. Only 10 anthocyanin compounds were identified based on pure standards: delphinidin-3-*O*-galactoside (Dp-3-*O*-Gal, peak 1), delphinidin-3-*O*-glucoside (Dp-3-*O*-Glc, peak 2), cyanidin-3-*O*-galactoside (Cy-3-*O*-Gal, peak 3), cyanidin-3-*O*-glucoside (Cy-3-*O*-Glc, peak 4), petunidin-3-*O*-glucoside co-eluting with cyanidin-3-*O*-arabinoside (Pt-3-*O*-Glc + Cy-3-*O*-Ara, peaks 5,6), peonidin-3-*O*-galactoside (Pn-3-*O*-Gal, peak 7), peonidin-3-*O*-glucoside co-eluting with malvidin-3-*O*-galactoside (Pn-3-O-Glc + Mv-3-*O*-Gal, peaks 8,9), and malvidin-3-O-glucoside (Mv-3-*O*-Glc, peak 10). These ten compounds are representative of the five major anthocyanidin classes (delphinidin, cyanidin, petunidin, peonidin, and malvidin) typically reported in highbush blueberry fruit, predominantly occurring as 3-galactoside, 3-glucoside, and 3-arabinoside conjugates. All ten compounds were detected across the studied genotypes, although the relative proportions of individual peaks varied considerably among cultivars and harvest years, as evidenced by the chromatographic profiles shown in Figure A1.

The sum of anthocyanin compounds, as determined by UPLC-PDA, was calculated for each blueberry genotype by summing the peak areas of the individual anthocyanins detected in the chromatograms and was quantified across all three harvest seasons (Table 1). In 2023, data were available for eight genotypes, excluding Safir. Lax exhibited the highest total anthocyanin content (678.34 mg/100 g FW), followed by Delicia (453.47 mg/100 g FW) and Pastel (420.51 mg/100 g FW), while Augusta recorded the lowest value (188.07 mg/100 g FW). In 2023, ‘Lax’ exhibited the highest total anthocyanin content (678.34 mg/100 g FW), being assigned to a separate statistical group (a′) and differing significantly from all other genotypes. ‘Delicia’ (453.47 mg/100 g FW) and ‘Pastel’ (420.51 mg/100 g FW) were grouped together (b′), without significant differences between them. ‘Prod’, ‘Simultan’, and ‘Vital’ formed a common statistical group (c′), while ‘Augusta’ and ‘Azur’ recorded the lowest values, without significant differences between them (d′), as indicated by overlapping letter groupings in Table 1.

In 2024, ‘Lax’ again showed the highest sum of anthocyanins (629.37 mg/100 g FW), followed by ‘Simultan’ (520.99 mg/100 g FW), ‘Pastel’ (497.74 mg/100 g FW), ‘Safir’ (482.33 mg/100 g FW), ‘Delicia’ (479.64 mg/100 g FW), ‘Augusta’ (475.57 mg/100 g FW), ‘Vital’ (398.83 mg/100 g FW), ‘Azur’ (372.01 mg/100 g FW), and ‘Prod’ (298.29 mg/100 g FW). In 2025, ‘Lax’ retained the highest content (596.86 mg/100 g FW), followed by ‘Pastel’ (492.78 mg/100 g FW) and ‘Simultan’ (440.79 mg/100 g FW), while ‘Azur’ (317.58 mg/100 g FW) and ‘Prod’ (300.24 mg/100 g FW) showed the lowest values. A general decreasing trend in total anthocyanin content from 2024 to 2025 was observed for most genotypes, with ‘Azur’ and ‘Prod’ remaining comparatively stable across both years. The consistently highest accumulation of anthocyanin in ‘Lax’ over all three harvest seasons highlights its potential as a high-anthocyanin genotype, whereas ‘Prod’ exhibited the lowest total anthocyanin content throughout the entire study period.

The individual anthocyanin composition of nine *V. corymbosum* genotypes was quantified by UPLC-PDA across three consecutive harvest seasons (2023, 2024, and 2025). The ten anthocyanin compounds identified were quantified on the basis of pure standards: All quantitative data are presented in Table 2.

Significant genotypic variation was observed in the detection and quantification of individual anthocyanins. Dp-3-*O*-Gal (peak 1) and Cy-3-*O*-Gal (peak 3) were detected in all nine genotypes across all three harvest years, with the exception of ‘Safir’, for which no data were available in 2023. By contrast, Cy-3-O-Glc (peak 4) and Mv-3-O-Glc (peak 10) were not detected in ‘Prod’ in any year. In ‘Azur’, Cy-3-O-Glc (peak 4) and Mv-3-O-Glc (peak 10) were absent across all three years, while Dp-3-O-Glc (peak 2) was detected in 2024 and 2025 but not in 2023. In ‘Augusta’, Dp-3-O-Glc (peak 2) and Cy-3-O-Glc (peak 4) were absent only in 2023, being detected in both subsequent years. ‘Safir’ was not analysed in 2023. Prod’ consistently exhibited a five-compound profile (peaks 1, 3, 5/6, 7, 8/9) across all three harvest years, with Dp-3-O-Glc (peak 2), Cy-3-O-Glc (peak 4), and Mv-3-O-Glc (peak 10) remaining below the detection limit in all years, indicating a qualitatively reduced and stable anthocyanin profile for this genotype. In ‘Azur’, the same five compounds were detected in 2023, while Dp-3-O-Glc (peak 2) became detectable in 2024 and 2025, suggesting a partial inter-annual variation in the anthocyanin profile of this genotype.

Dp-3-*O*-Gal (peak 1) showed the widest inter-genotype range of all individual compounds. In 2023, values ranged from 19.89 mg/100 g FW (‘Augusta’) to 93.60 mg/100 g FW (‘Lax’). In 2024, the range extended from 29.71 mg/100 g FW (‘Safir’) to 85.11 mg/100 g FW (‘Lax’), with most genotypes recording higher values than in 2023, the exceptions being ‘Delicia’ (45.01 to 34.52 mg/100 g FW) and ‘Prod’ (40.56 to 33.89 mg/100 g FW). In 2025, Dp-3-*O*-Gal decreased in all genotypes relative to 2024, with ‘Lax’ retaining the highest value (63.90 mg/100 g FW) and ‘Augusta’ recording the lowest among the profiled genotypes (26.56 mg/100 g FW). Statistical analysis revealed significant differences among several genotypes within each year for Dp-3-O-Gal, as indicated by distinct and overlapping letter groupings in Table 2. In all three years, ‘Lax’ was consistently assigned to a separate statistical group, recording significantly higher concentrations than all other genotypes, while several other genotypes shared overlapping statistical groups, indicating no significant differences among them. Dp-3-*O*-Glc (peak 2) was absent in ‘Prod’ for all three harvest years. In ‘Augusta’ and ‘Azur’, this compound was not detected in 2023 but was present in 2024 and 2025, indicating an inter-annual variation in its accumulation in these two genotypes. Among the genotypes in which it was detected, values in 2023 ranged from 16.18 mg/100 g FW (‘Vital’) to 36.32 mg/100 g FW (‘Lax’). In 2024, concentrations generally increased, with ‘Lax’ (33.96 mg/100 g FW), ‘Pastel’ (32.27 mg/100 g FW), and ‘Safir’ (30.23 mg/100 g FW) showing the highest values, without significant differences among them (groups A, A,B, and A,B,C, respectively, Table 2). In 2025, ‘Lax’ (32.06 mg/100 g FW) and ‘Pastel’ (31.01 mg/100 g FW) recorded the highest concentrations of Dp-3-*O*-Glc, assigned to the top statistical group (a), without significant differences between them. ‘Safir’ (28.16 mg/100 g FW) was assigned to an overlapping group (a,b), not differing significantly from ‘Lax’ and ‘Pastel’, while the remaining genotypes recorded significantly lower values (Table 2).

Cy-3-*O*-Gal (peak 3) followed a distribution pattern relatively similar to Dp-3-*O*-Gal. In 2025, ‘Lax’ (32.06 mg/100 g FW) and ‘Pastel’ (31.01 mg/100 g FW) recorded the highest concentrations of Dp-3-*O*-Glc, assigned to the top statistical group (a), without significant differences between them. ‘Safir’ (28.16 mg/100 g FW) was assigned to an overlapping group (a,b), not differing significantly from ‘Lax’ and ‘Pastel’, while the remaining genotypes recorded significantly lower values (Table 2). In 2024, concentrations increased for most genotypes, particularly for ‘Simultan’ (25.72 to 45.29 mg/100 g FW) and ‘Azur’ (22.78 to 39.35 mg/100 g FW), while ‘Delicia’ decreased (32.16 to 27.42 mg/100 g FW). In 2025, values decreased in all genotypes, ranging from 20.23 mg/100 g FW (‘Delicia’) to 49.77 mg/100 g FW (‘Lax’). Cy-3-O-Glc (peak 4) was absent in ‘Azur’ and ‘Prod’ for all three harvest years, and was also not detected in ‘Augusta’ and ‘Safir’ in 2023. Among the genotypes and years in which it was detected, concentrations were generally lower compared to the other individual anthocyanins, ranging from 12.66 mg/100 g FW (‘Vital’, 2023) to 16.94 mg/100 g FW (‘Lax’, 2024), with limited inter-genotype variability (Table 2). The co-eluting fraction Pt-3-*O*-Glc + Cy-3-*O*-Ara (peaks 5,6) was detected in all genotypes and years. In 2023, concentrations ranged from 13.05 mg/100 g FW (‘Augusta’) to 33.33 mg/100 g FW (‘Lax’). In 2024, values increased for most genotypes and the inter-genotype range widened (13.58 mg/100 g FW in ‘Prod’ to 34.57 mg/100 g FW in ‘Lax’). In 2025, concentrations generally decreased, with ‘Prod’ recording the lowest value across all three years for this fraction (14.16 mg/100 g FW) and ‘Lax’ retaining the highest value (31.05 mg/100 g FW).

Pn-3-*O*-Gal (peak 7) was detected in all genotypes and years, with the exception of ‘Safir’ for which no data were available in 2023. Concentrations were consistently lower than those of the delphinidin and cyanidin galactosides for all genotypes and years (Table 2). In 2023, values ranged from 12.02 mg/100 g FW (‘Augusta’) to 27.75 mg/100 g FW (‘Lax’). In 2024, concentrations increased across most genotypes, ranging from 15.81 mg/100 g FW (‘Safir’) to 27.48 mg/100 g FW (‘Lax’). In 2025, values decreased in all genotypes compared to 2024, ranging from 12.48 mg/100 g FW (‘Delicia’) to 22.25 mg/100 g FW (‘Lax’), with significant differences among several genotypes as indicated by the overlapping letter groupings in Table 2. The co-eluting fraction Pn-3-O-Glc + Mv-3-*O*-Gal (peaks 8,9) was detected in all genotypes and years in which analysis was performed, with the exception of ‘Safir’, for which no data were available in 2023. In 2023, ‘Lax’ recorded the highest concentration (54.53 mg/100 g FW) and ‘Augusta’ the lowest (16.80 mg/100 g FW). In 2024, the co-eluting fraction Pn-3-*O*-Glc + Mv-3-*O*-Gal ranged from 29.71 mg/100 g FW (‘Delicia’) to 68.65 mg/100 g FW (‘Azur’), with significant differences among genotypes as indicated by the statistical groupings in Table 2. In 2025, ‘Lax’ recorded the highest concentration (36.73 mg/100 g FW), while ‘Delicia’ showed the lowest value (21.22 mg/100 g FW), with several genotypes sharing overlapping statistical groups (Table 2). ‘Azur’ showed the most pronounced inter-annual variation for this compound fraction across the entire dataset (20.15 to 68.65 to 23.86 mg/100 g FW).

Mv-3-O-Glc (peak 10) showed the widest concentration range among all individual anthocyanins. Mv-3-O-Glc (peak 10) was absent in ‘Azur’ and ‘Prod’ for all three harvest years, while in ‘Safir’ it was absent in 2023 but present in 2024 (86.12 mg/100 g FW) and 2025 (64.11 mg/100 g FW). Among genotypes in which it was detected, ‘Safir’ recorded by far the highest concentration in 2024 (86.12 mg/100 g FW), substantially exceeding the next highest value of 80.90 mg/100 g FW recorded in ‘Lax’ in the same year. In 2025, ‘Safir’ again recorded the highest Mv-3-*O*-Glc concentration (64.11 mg/100 g FW), followed by ‘Lax’ (52.87 mg/100 g FW). The lowest detected values were recorded in ‘Simultan’ in 2023 (21.87 mg/100 g FW) and ‘Augusta’ in 2025 (26.69 mg/100 g FW). Among genotypes in which Mv-3-*O*-Glc was detected, ‘Vital’ recorded 59.31 mg/100 g FW in 2024 and 31.79 mg/100 g FW in 2025, without significant differences from several other genotypes (Table 2).

**Figure 4:**
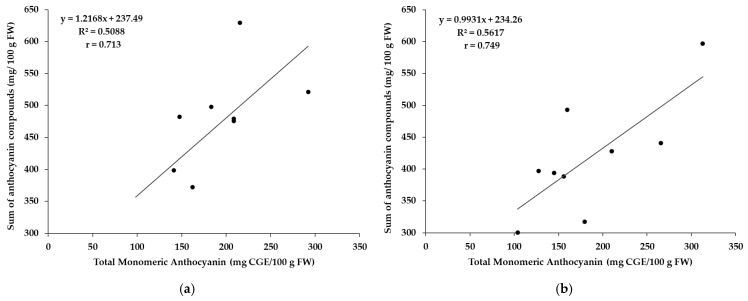
Correlation between Total Monomeric Anthocyanin content (TMA, pH differential method) and sum of anthocyanin compounds by UPLC-PDA anthocyanin sum in the *V. corymbosum* genotypes in 2024 (**a**) and 2025 (**b**). Solid line: linear regression. Pearson’s r and R^2^ indicated per panel.

Correlation analysis between TMA and the sum of anthocyanin compounds (Figure 4) indicates a moderately positive relationship in both years (r = 0.713, R^2^ = 0.509 in 2024; r = 0.749, R^2^ = 0.562 in 2025), as reflected by the linear regression equations displayed in Figure 4. The regression slopes close to unity (2024: y = 0.972x + 212.45; 2025: y = 0.993x + 234.26) suggest a proportional relationship between the two quantification methods, but the scatter around the regression line indicates that they cannot be used interchangeably for absolute quantification.

## 3. Discussion

Understanding the relationship between genotype and major fruit quality characteristics—including fruit size, firmness, soluble solids content, titratable acidity, and phytochemical composition—is essential for the evaluation and improvement of highbush blueberries (*Vaccinium corymbosum* L.). These attributes determine the agronomic, commercial, sensory, and nutritional value of the blueberry fruit. Fruit size and firmness are critical for harvest efficiency, market acceptance, transportability, and postharvest shelf life, while the balance between SSC and TA strongly influences sweetness, flavor intensity, taste and consumer preference [24,25,26]. In parallel, the accumulation of anthocyanins, phenolic compounds, and other bioactive metabolites largely defines the antioxidant capacity and health-promoting properties of blueberries.

This research evaluates how genetic background and inter-annual environmental variability influence the physical, chemical, and phytochemical characteristics of blueberry (*Vaccinium corymbosum* L.) fruit, with particular emphasis on fruit weight, size, firmness, total soluble solids, titratable acidity, total phenolic content, anthocyanins, and antioxidant activity. By assessing nine genotypes over three consecutive harvest seasons, the study provides a comprehensive analysis of the interactions between genotype and annual climatic conditions and their contribution to the phenotypic expression of characteristics that determine commercial quality, sensory attributes, and functional value. The results offer important insights into the stability of fruit quality traits across years and enable the identification of genotypes combining superior agronomic performance with consistently high levels of health-promoting phytochemicals.

### 3.1. Genotypic and Inter-Annual Variation in Fruit Physical Characteristics

Significant genotypic differences in fruit weight, polar and equatorial dimensions, and firmness were observed across all three harvest seasons, confirming the predominant role of genetic background in determining the physical characteristics of blueberry fruit. ‘Augusta’ showed the highest fruit weight and the largest equatorial dimensions in 2023, while ‘Lax’ recorded the smallest fruit weight in the same year, in line with previous reports on the wide within-species variation for this parameter in *V. corymbosum*. Kim et al. [30] reported fruit weights ranging from 0.9 to 3.6 g across 45 northern highbush cultivars grown in Korea, a range that includes the variation observed in this study. The biometrical characterisation of Romanian cultivars performed by Hera et al. [9] reported a fruit weight of 3.1 g for ‘Delicia’. The lower value recorded for this cultivar in 2024 in the present study (1.74 ± 0.25 g) is likely attributable to the marked inter-annual climatic variability confirmed as the dominant source of variation (η^2^p year = 0.798), combined with differences in plant age and crop load. Notably, ‘Delicia’ reached 2.56 ± 0.25 g in 2025, approaching the previously reported value, which supports the conclusion that the lower 2024 values reflect seasonal rather than cultivar-specific constraints.

A significant decrease in fruit weight in 2024 relative to 2023 was observed only for ‘Augusta’, while most other genotypes remained stable. A marked increase in 2025 was observed across all genotypes, most notably in ‘Azur’ and ‘Augusta’, emphasizing the importance of the year effect on physical fruit characteristics, as confirmed by two-way ANOVA. Such inter-annual variations are well documented for *V. corymbosum* and have been attributed to the different climatic growing conditions during fruit development, plant age, and crop load [24]. It has been demonstrated that year × harvest interactions significantly affected the majority of the phenotypic parameters for five blueberry cultivars grown at two locations over two years, with clonal plant replicates being the most important single source of variability [17]. The marked inter-annual variation confirmed by two-way ANOVA in the present study highlights the necessity of multi-year evaluations for comprehensive characterisation of cultivar performance.

Fruit firmness showed both genotype- and year-dependent variation. In 2025, ‘Prod’, ‘Simultan’, and ‘Vital’ recorded the highest firmness values, especially in 2025, without significant differences among them, suggesting excellent potential for long-distance transport and extended shelf life. Firmness is a parameter of direct commercial relevance, influencing postharvest handling and shelf-life [17]. Consequently, these three genotypes may represent genotypes of interest for fresh-market and extended-storage applications.

### 3.2. Genotypic and Inter-Annual Variation in Fruit Chemical Characteristics

Total soluble solids and titratable acidity are key determinants of sweetness, tartness, and overall consumer acceptability. TSS exhibited significant genotype- and year-dependent variation as confirmed by two-way ANOVA (Table A1). The TSS range observed in the present study (9 to 16% Brix) is comparable to values reported for 45 Korean-grown highbush cultivars (8.3–14.3% Brix) [30] and for Romanian cultivars (8.93–19.3% Brix) [28], confirming that the genotypes studied here fall within the typical range for the species. The marked decrease observed for ‘Augusta’ in 2024 likely reflects the sensitivity of soluble solids accumulation to growing-season conditions, particularly temperature and solar radiation during fruit maturation, which are known to directly influence sugar biosynthesis and accumulation in blueberry fruit [24]. The absence of a consistent inter-annual pattern for most other genotypes suggests that TSS is primarily under genetic control in these cultivars, with environmental conditions acting as a secondary modulating factor [24,26].

The predominant role of genetic background in determining titratable acidity was reflected in the exceptionally high proportion of variance explained by the two-way ANOVA model, suggesting that acidity is a highly heritable and genetically stable trait in the Romanian cultivars. This pattern is directly relevant to fruit flavour, since the soluble solids–acidity balance determines the sweet–sour ratio that governs consumer acceptability [17]. The notably low TA observed for ‘Augusta’ and ‘Azur’ in 2023, combined with TSS values comparable to other genotypes, suggests a more balanced and potentially sweeter-perceived flavour profile for these genotypes under favourable growing conditions, whereas ‘Delicia’, with consistently high acidity across all three years, would be expected to present a distinctly tart flavour profile. The TA values obtained in this study (0.23–1.14 g malic acid/100 g FW) are comparable to those reported for international highbush cultivars (0.8–3.6% citric acid equivalents) [30], once differences in expression units are considered.

### 3.3. Phenolic Content, Monomeric Anthocyanins, and Antioxidant Activity Across Genotypes and Harvest Years

The TPC showed considerable variation among genotypes and harvest year, with the lowest values observed in ‘Prod’ (2025) and the highest in ‘Augusta’ (2025). These findings are in accordance with the literature for blueberry (*Vaccinium corymbosum* L.) fruit. Kraujalyte et al. [31] reported TPC values of 0.85–2.81 mg GAE/mL for juices of various *V. corymbosum* genotypes. When expressed on a fresh weight basis and accounting for typical juice yield, these values are broadly compatible with the range observed in the present study, although direct comparison should be made with caution due to differences in matrix and extraction methodology. Lachowicz-Wiśniewska [27] observed cultivar-dependent variation in a panel of 14 northern highbush cultivars, confirming that TPC is primarily a genotype-controlled parameter. The remarkably low and stable TPC of ‘Prod’ across both years (200.17 and 183.82 mg GAE/100 g FW in 2024 and 2025, respectively) stands out as a cultivar-specific characteristic of this genotype and warrants further investigation into the genetic and biochemical mechanisms underlying phenolic biosynthesis.

The TMA content exhibited substantial variation among genotypes and harvest years. ‘Prod’ consistently recorded the lowest values, whereas ‘Lax’ showed the highest TMA in 2025, with a marked increase relative to the previous year. In contrast, ‘Simultan’ exhibited the highest TMA in 2024. The observed range is comparable to TMA values previously reported for highbush blueberry cultivars using the same AOAC 2005.02 methodology [30] who found total anthocyanin contents ranging from 167.6 to 677.8 mg CGE/100 g FW in 45 blueberry cultivars grown in Korea. In another study, determined values of 93.1–235.4 mg CGE/100 g FW were reported for selected *Vaccinium* species [1].

The generally higher TMA of ‘Lax’ is interesting, since it is closely related to both ‘Simultan’, and ‘Pastel’, as all three have been obtained by open pollination of ‘Spartan’ [9]. The phytochemical differences observed among these three half-sibling genotypes indicate that anthocyanin accumulation capacity segregates independent of the common maternal genetic background and is likely determined by allelic variation contributed by different, unknown pollen donors.

Antioxidant activity showed substantial variation among genotypes and harvest years, with the lowest values consistently observed in ‘Prod’ and the highest in ‘Simultan’. ‘Simultan’ consistently maintained the highest for antioxidant capacity in both measured years, while ‘Safir’ ranked second in 2025.

The ranking pattern of antioxidant activity closely followed those observed for TPC and TMA, suggesting that phenolic compounds and anthocyanins are the primary contributors to radical-scavenging capacity in the studied genotypes. A numerical decrease in TPC and antioxidant activity between 2024 and 2025 was observed for several genotypes, while ‘Augusta’, ‘Safir’, and ‘Lax’ showed the opposite trend. This divergent inter-annual behavior is consistent with a genotype × environment interaction, which was formally confirmed by the two-way ANOVA (Table A1), and is in agreement with previous studies describing this interaction as a decisive determinant of blueberry chemotype [26]. It was also demonstrated that genotype is the primary driver of antioxidant capacity and phenolic content in *V. corymbosum*, with cultivation system and irrigation regime exerting secondary, cultivar-specific modulating effects [26]. The stability of ‘Simultan’ and ‘Prod’ rankings across years, with ‘Simultan’ being the highest, and ‘Prod’ the lowest, suggests that these genotypes express a genetically fixed phenolic accumulation capacities, relatively insensitive to inter-annual variation. These results indicate that genotypes with elevated phenolic and anthocyanin contents also possess superior radical-scavenging capacity, supporting numerous studies linking blueberry polyphenols with oxidative stress reduction and potential protective effects against chronic diseases.

### 3.4. Anthocyanin Content and Qualitative Profile

Anthocyanins are the dominant pigments in blueberry fruit and represent one of the most important classes of bioactive compounds. Ten anthocyanins were identified using UPLC-PDA analysis, representing the major delphinidin, cyanidin, petunidin, peonidin, and malvidin glycosides typically reported fingerprint of *V. corymbosum* fruit [1,27,31,32]. This qualitative profile is consistent with previous investigations of cultivated blueberry fruit and confirms that the Romanian genotypes possess the characteristic anthocyanin spectrum of the species. Lätti et al. identified a qualitatively identical set of derivatives using the same UPLC approach adopted in the present study, providing direct methodological comparability [32].

The quantitative differences among cultivars were substantial. ‘Lax’ consistently accumulated the highest anthocyanin levels in all three years, reaching 678.34 mg/100 g FW in 2023 and remaining above 596 mg/100 g FW in 2025. ‘Pastel’ and ‘Simultan’ also maintained high anthocyanin concentrations, whereas ‘Prod’ and ‘Azur’ exhibited the lowest values. The high anthocyanin accumulation observed in ‘Lax’ indicates a strong genetic predisposition for enhanced flavonoid biosynthesis and identifies this cultivar as an important source of natural pigments and antioxidant compounds.

**Figure A2:**
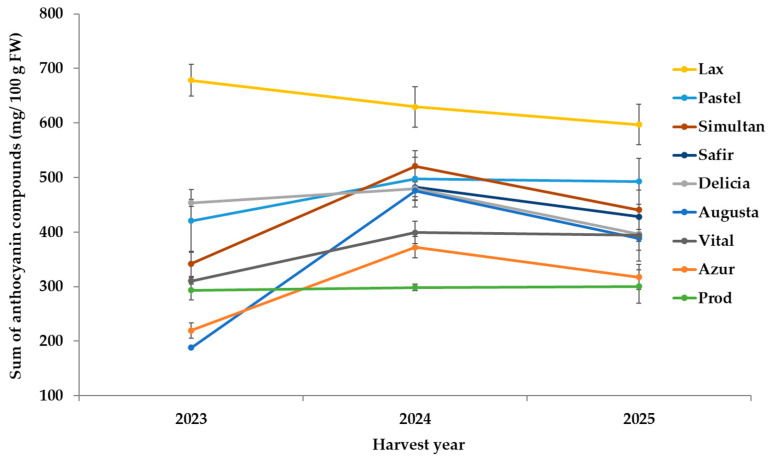
Inter-annual variation in sum of anthocyanin compounds (mg/100 g FW) in the *V. corymbosum* genotypes for harvest years 2023, 2024, and 2025. Safir was not determined in 2023.

The inter-annual trajectories of all nine genotypes are illustrated in Figure A2. Within each harvest year, one-way ANOVA revealed significant differences among genotypes for the sum of anthocyanin compounds (Table 1). ‘Lax’ consistently maintained the highest anthocyanin levels across all three years, while ‘Azur’ and ‘Prod’ recorded the lowest values in all measured seasons, without significant differences between these two genotypes in any year (Table 1).

Liu et al. (2025) identified malvidin 3-*O*-galactoside, delphinidin 3-*O*-galactoside, delphinidin 3-*O*-arabinoside, malvidin 3-*O*-arabinoside, and petunidin 3-*O*-galactoside as the five most abundant individual anthocyanins in 26 Chinese highbush cultivars, a composition consistent with the quantitative profile observed here, where galactoside and arabinoside conjugates constituted the predominant fraction [8].

Interestingly, the total anthocyanin content determined by UPLC-PDA was consistently higher than the total monomeric anthocyanin values obtained by the pH differential method. This difference arises because chromatographic quantification accounts for individual anthocyanins using calibration against external standards, whereas spectrophotometric methods estimate total pigments relative to a single reference compound and may underestimate complex anthocyanin mixtures. Additionally, UPLC-PDA quantification is performed on freeze-dried extracts, which can result in enhanced extraction efficiency and recovery of anthocyanins [27,30]. The inter-annual decrease in anthocyanin content may be related to differences in temperature and solar radiation during fruit ripening, given that high temperatures during maturation are known to inhibit anthocyanin biosynthesis by reducing the expression of key pathway genes [24,33]. The greater decrease in values for ‘Delicia’ (from 479.64 to 396.75 mg/100 g FW) compared to ‘Lax’ (from 629.37 to 596.86 mg/100 g FW) may indicate that the regulation of anthocyanin biosynthesis is different in the two cultivars in response to seasonal variation [17,24,26].

The qualitative anthocyanin profile found in all nine *V. corymbosum* genotypes, comprising the characteristic aglycones delphinidin, cyanidin, petunidin, peonidin, and malvidin predominantly present as 3-galactoside, 3-glucoside, and 3-arabinoside conjugates, is consistent with the established anthocyanin fingerprint of highbush blueberry [31,32]. The Dp-3-*O*-Gal and Cy-3-*O*-Gal were the most abundant individual compounds in all genotypes across all three years, in agreement with previous blueberry berry studies on [8,27]. This pattern contrasts with that reported for *V. myrtillus*, in which galactosides, glucosides, and arabinosides contributed comparably to the total anthocyanin profile (32%, 39%, and 29%, respectively), reflecting the difference in glycosylation preference between the two species [32].

Dp-3-*O*-Gal was the compound showing the largest inter-genotype variability range, ranging from 19.89 mg/100 g FW in ‘Augusta’ (2023) to 93.60 mg/100 g FW in ‘Lax’ (2023), a 4.7-fold difference. A similarly wide within-species variation of approximately 4.5-fold was reported for total anthocyanin contents across 179 *V. myrtillus* individuals, where genetic origin was identified as the primary source of variation [32]. The 2023 to 2024 increase in Dp-3-*O*-Gal observed for most genotypes (‘Simultan’: 29.23 to 53.10 mg/100 g FW; ‘Augusta’: 19.89 to 38.76 mg/100 g FW) is consistent with the reported stimulatory effect of light intensity on anthocyanin accumulation [16,33], given that *V. corymbosum* anthocyanin biosynthesis is known to be responsive to solar radiation and temperature conditions during the fruit maturation window [26]. The decrease observed in 2025 for all genotypes is similar to the environmental modulation of anthocyanin content reported in *V. myrtillus* populations, where temperature and light were identified as the main environmental drivers [32].

Prod’ consistently exhibited a qualitatively reduced anthocyanin profile for all three harvest years, with Dp-3-*O*-Glc (peak 2), Cy-3-*O*-Glc (peak 4), and Mv-3-*O*-Glc (peak 10) remaining below the detection limit in all years. In ‘Azur’, Cy-3-*O*-Glc (peak 4) and Mv-3-*O*-Glc (peak 10) were absent across all three years, while Dp-3-*O*-Glc (peak 2) was not detected in 2023 but was present in 2024 and 2025. A comparable phenomenon of reduced or absent glucoside biosynthesis at the individual plant level was documented in *V. myrtillus*, where seven exceptional individuals out of 179 displayed glucoside proportions below 4%, a pattern attributed to genetic variation in glucosyltransferase activity [32]. The constitutive nature of this reduction in ‘Azur’ and ‘Prod’ across all three harvest years, and across independently prepared extracts, indicates that this is a stable genotypic trait rather than a seasonal response. A similar cultivar-specific variation in the completeness of the anthocyanin profile was noted among highbush blueberry accessions, the absence of specific conjugates being attributed to below-detection-limit glycosyltransferase activity rather than pathway absence [27,34].

Mv-3-*O*-Glc (peak 10) showed the greatest range of all individual compounds and was distinguished by the high concentrations recorded in ‘Safir’ in 2024 (86.12 mg/100 g FW) and 2025 (64.11 mg/100 g FW). These values exceed those reported for Mv glycosides in other *V. corymbosum* cultivars [8,31], and are close to the values of the Mv glycoside contents found in some *V. myrtillus* individuals from Finland, where bilberry individuals contained Mv amounts (>90 mg/100 g FW) comparable to those found in lowbush blueberry (*V. angustifolium*), characterised by a high proportion of Mv derivatives [32]. The disproportionately high Mv-3-*O*-Glc accumulation in ‘Safir’ relative to the other Romanian cultivars may reflect an elevated activity of the late-pathway enzymes involved in anthocyanin B-ring methylation and glucosylation, specifically *O*-methyltransferase and UDP-glucose:flavonoid 3-*O*-glucosyltransferase, as proposed for Mv-rich *Vaccinium* genotypes [20]. This enzymatic capacity is likely genetically encoded, given the stability of the high Mv-3-*O*-Glc proportion in ‘Safir’ across both measured harvest years, and may reflect allelic variation at loci controlling the late steps of the anthocyanin pathway. Environmental modulation cannot be excluded as a secondary factor, since temperature and light conditions during fruit maturation are known to influence the expression of anthocyanin biosynthetic genes in *Vaccinium* [12,13,29]; however, the consistent ranking of ‘Safir’ as the highest Mv-3-*O*-Glc accumulator across years suggests that genetic background is the primary determinant of this trait in the Romanian cultivar set. The disproportionate contribution of Mv-3-*O*-Glc to the total anthocyanin profile of ‘Safir’ (approximately 36 to 43% of the quantified sum of anthocyanins depending on the year) is a distinctive qualitative feature that sets this cultivar apart from all others in the Romanian set, in which delphinidin and cyanidin galactosides dominated. This pattern may reflect an elevated O-methyltransferase and glucosyltransferase activity in the late steps of the anthocyanin pathway in ‘Safir’, as proposed for Mv-rich *Vaccinium* [27].

In 2024, the average proportional contributions of the five anthocyanidin classes to the total quantified sum of anthocyanins were approximately: delphinidin, 28 to 35%; cyanidin, 22 to 28%; the co-eluting petunidin–cyanidin arabinoside fraction, 11 to 16%; peonidin, 8 to 12%; and malvidin, 15 to 40%. The lower proportional contribution of malvidin in most Romanian cultivars compared to ‘Safir’ mirrors inter-cultivar differences in Mv proportion reported previously for European highbush blueberry panels [27,31]. By contrast, Lätti et al. [32] found that the proportion of Mv was 14 to 19% higher in *V. corymbosum* than in *V. myrtillus* on average, reflecting the greater capacity for late-pathway *O*-methylation in the cultivated species. This trend is confirmed by the present results for most genotypes, with ‘Safir’ representing an extreme within-species expression of this tendency.

Cy-3-*O*-Gal followed a distribution pattern similar to Dp-3-*O*-Gal across genotypes and years, with ‘Lax’ consistently highest (70.36 mg/100 g FW in 2023; 69.57 n 2024; 49.77 mg/100 g FW in 2025) and ‘Augusta’ or ‘Delicia’ lowest depending on the year. The ratio of Dp to Cy galactosides was relatively stable within individual genotypes across years but differed among genotypes, ranging from approximately 1.0 in ‘Simultan’ and ‘Vital’ to 1.5 in ‘Lax’. In the study of Lätti et al. [32], it was mentioned that the Dp:Cy ratio is largely under genetic control, through the flavonoid 3′,5′-hydroxylase to flavonoid 3′-hydroxylase activity ratio, which determines the partition between the delphinidin and cyanidin branches of the pathway. The genotype-specificity of this ratio in our study is consistent with that conclusion and confirms that the relative hydroxylation capacity at the B-ring is genotype-encoded in the Romanian *V. corymbosum* cultivars, in agreement with a similar study [27].

The co-eluting fractions of Pn-3-*O*-Glc and Mv-3-*O*-Gal (peaks 8,9) showed the most pronounced inter-annual variation in ‘Azur’, increasing from 20.15 mg/100 g FW in 2023 to 68.65 mg/100 g FW in 2024, then decreasing to 23.86 mg/100 g FW in 2025, a 3.4-fold increase from 2023 to 2024. Such an amplitude of seasonal variation in a single compound fraction is larger than typically reported in multi-year blueberry studies [24,26], suggesting that in ‘Azur’ the methylation steps producing peonidin and malvidin derivatives are particularly sensitive to inter-annual climatic variation. This interpretation is consistent with the observation that temperature and light act on specific steps of the anthocyanin pathway rather than uniformly across all compounds [32], and with the genotype-dependent sensitivity to seasonal conditions [26].

The general increase from 2023 to 2024 followed by a decrease in 2025 observed for most genotypes and compounds contrasts with the decrease recorded in ‘Lax’ and ‘Prod’ across all three years. ‘Lax’ maintained the highest concentrations of the majority of individual compounds across all years despite an absolute decrease, suggesting that its genotype-encoded biosynthetic capacity is sufficiently high to remain superior to other cultivars even under less favourable seasonal conditions. This type of stable high-accumulator phenotype was described for *V. myrtillus* individuals with constitutively high anthocyanin contents, attributed to genetic rather than environmental origin [32]. The stability of ‘Prod’ as the consistently lowest-accumulating genotype across all years and compounds similarly indicates that its anthocyanin biosynthetic output is genetically constrained and not responsive to seasonal variation. Together, these observations support the conclusion that genotype is the primary determinant of individual anthocyanin profile in *V. corymbosum*, with season acting as a modulating factor whose magnitude and direction of effect are themselves cultivar-dependent, in agreement with the genotype × environment framework established for *Vaccinium* polyphenols [24,26].

### 3.5. Relationship Between Genetic Background and Phytochemical Characteristics

The marked differences observed among blueberry genotypes support the hypothesis that the genetic background is a major determinant of fruit phytochemical composition. Previous molecular analyses using SRAP markers revealed substantial genetic diversity among the Romanian genotypes, with ‘Pastel’ occupying a particularly distinct genetic position. The present results demonstrate that this genetic diversity is reflected in contrasting phenotypes, especially with respect to anthocyanin accumulation, antioxidant activity, and fruit quality.

Certain genotypes combined favorable horticultural and nutritional characteristics. ‘Simultan’ exhibited high antioxidant activity and elevated monomeric anthocyanin content; ‘Safir’ showed consistently high phenolic content and antioxidant capacity; ‘Lax’ was distinguished by its high anthocyanin accumulation; and ‘Augusta’ combined large fruit size, high soluble solids, and elevated phenolic content. These complementary trait profiles provide valuable information for breeding programs aimed at combining fruit quality, storability, and nutritional functionality.

### 3.6. Future Research Directions: Implications for Nutraceutical Valorisation and Breeding

The identification of genotypes with superior phytochemical profiles has direct implications for both commercial production and breeding. Genotypes such as ‘Lax’, ‘Simultan’, ‘Safir’, and ‘Augusta’ represent promising genetic resources for developing new blueberry varieties with enhanced bioactive compound profiles and nutraceutical value. By contrast, ‘Prod’ exhibited the lowest phenolic content while achieving the highest firmness in 2025, a trait of importance for mechanical harvesting and extended fresh-market shelf-life. The combination of large fruit size, high TSS, and low acidity observed in ‘Augusta’ and ‘Azur’ in 2023 and 2025 further identifies these cultivars as promising candidates for fresh consumption, where organoleptic quality is prioritized over phytochemical richness. In addition, these genotypes may be particularly suitable for the production of functional foods, natural colorants, and antioxidant-rich ingredients for the food and nutraceutical industries.

Future research should integrate metabolomic, transcriptomic, and genomic approaches to elucidate the molecular mechanisms underlying anthocyanin biosynthesis and phenolic accumulation in these Romanian genotypes. Multi-location trials would also be valuable to assess the stability of these traits under different environmental conditions and to better characterize genotype × environment interactions.

## 4. Materials and Methods

### 4.1. Blueberry Samples

Blueberry fruits (*V. corymbosum* L.) from nine cultivars—‘Lax’, ‘Prod’, ‘Vital’, ‘Azur’, ‘Simultan’, ‘Delicia’, ‘Pastel’, ‘Safir’, and ‘Augusta’—were collected at the ripening stage during the years 2023, 2024 and 2025 (June to August), from Romania systematic blueberry breeding at the Research Institute for Fruit Growing Pitești-Mărăcineni, Romania.

### 4.2. Chemicals

Methanol, acetonitrile and formic acid were sourced from Honeywell Riedel-de Haën (Seelze, Germany). Sodium hydroxide solution (0.1 N) was supplied by Chimreactiv S.R.L. (Bucharest, Romania), while anhydrous sodium carbonate came from Lach-Ner, s.r.o. (Neratovice, Czech Republic). Gallic acid was purchased from Roth GmbH (Altensteig, Germany). Folin & Ciocalteu’s phenol reagent (2 N) and DPPH (1,1-diphenyl-2-picrylhydrazyl) were provided by Sigma-Aldrich Chemie GmbH (Steinheim, Germany). Trolox (6-hydroxy-2,5,7,8-tetramethylchroman-2-carboxylic acid) was acquired from Acros Organics, Fisher Scientific (Geel, Belgium). Hydrochloric acid (37%) was from Carl Roth (Karlsruhe, Germany).

Anthocyanin standards, cyanidin-3-*O*-galactoside, cyanidin-3-*O*-glucoside, cyanidin-3-*O*-arabinoside, delphinidin-3-*O*-galactoside, delphinidin-3-*O*-glucoside, petunidin-3-*O*-glucoside, malvidin-3-*O*-galactoside, malvidin-3-*O*-glucoside, peonidin-3-*O*-galactoside and peonidin-3-*O*-glucoside were from Extrasynthese (Genay, France); gallic acid was purchased from Roth GmbH (Altensteig, Germany).

Ultrapure water with a resistivity of 18.2 MΩ·cm at 25 °C was prepared using a Milli-Q water purification system (Merck Millipore, Darmstadt, Germany).

### 4.3. Determination of Physicochemical Characteristics of Blueberry Fruits

The analysis was performed on fresh blueberry fruits and covered a range of physical and chemical parameters, including average fruit weight (AFW), fruit dimensions, firmness, dry matter content (DM), total soluble solids (TSS), and titratable acidity (TA), with each parameter measured in 7–10 replicates.

#### 4.3.1. Physical Characteristics

The average fruit weight (AFW) was calculated by weighing 10 fruits using a precision balance (PS 6000.R2, Partner Corporation, Bucharest, Romania) and dividing the total weight by the number of fruits.

Polar (height, h) and equatorial (transversal diameter, D) dimensions were measured in millimeters using a Parkside HG00962A digital caliper (OWIM GmbH & Co. KG, Neckarsulm, Germany), following established protocols [35,36].

Firmness was determined using a 53205 Turoni digital penetrometer (Forli, Italy) equipped with a 3 mm piston, and the results reported as Newtons (N).

#### 4.3.2. Chemical Characteristics

Dry Matter Content (DM) was determined by drying 1 g of sample at 105 °C until constant weight (UN110, Memmert, Germany).

Total Soluble Solids (TSS) was measured with a DR301-95 digital refractometer (Krüss, Germany) and expressed as % Brix.

Titratable Acidity (TA) was analyzed using AOAC 942.15: a 5 g sample was mixed with 100 mL distilled water, titrated to pH 8.1 with 0.1 N NaOH using the TitroLine Easy titrator (Fisher Scientific, Schwerte, Germany), and expressed as g malic acid equivalents/100 g FW.

### 4.4. Extraction of Phenolic Compounds

For phenolic extraction, 1 g of fresh blueberries was ground, mixed with 10 mL of 70% aqueous methanol, and incubated overnight in the dark [36]. The mixture was shaken at 500 rpm for 1 h and centrifuged at 5000 rpm at 4 °C for 10 min. The supernatant was collected, and the residue re-extracted twice with 10 mL of 70% methanol. Extracts were combined and the volume of each sample was adjusted to 30 mL prior to the determination of the total phenolic content and DPPH analysis.

### 4.5. Determination of Total Phenolic Content (TPC)

The TPC was determined spectrophotometrically using the Folin–Ciocâlteu method, as described in [37]. The extracts were diluted at ratios of 1:50 and 1:10 with 60% ethanol. Aliquots of 10–50 µL of extract were mixed with 2.5 mL of water-diluted Folin–Ciocâlteu reagent (2 N, 1/10). After incubation at room temperature for 2 min, 2 mL of Na_2_CO_3_ solution (75 g/L) was added. The mixture was incubated for 15 min at 50 °C in a water bath and then cooled in an ice-water bath. The specific absorbance at 760 nm was immediately measured using a SPECORD 210 Plus UV–Vis spectrophotometer (Analytik Jena, Jena, Germany).

The TPC of the extracts was quantified using a gallic acid calibration curve (y = 0.0112x + 0.0632, R^2^ = 0.9969), and results were expressed as milligrams of gallic acid equivalents per gram of fresh weight (mg GAE/g FW). All measurements were performed in triplicate.

### 4.6. DPPH (2,2-Diphenyl-1-Picrylhydrazyl) Radical Scavenging Assay

The DPPH radical scavenging assay was performed following the method of Bujor et al. (2016), with modifications [18]. To 200 µL diluted extracts (1:50, 1:10), we added 2 mL of a 0.2 mM solution of DPPH in 60% ethanol. The mixture was incubated for 30 min under constant magnetic stirring in the dark at room temperature.

The absorbance at 515 nm was measured with a UV–Vis spectrophotometer (SPECORD 210 Plus, Analytik Jena, Jena, Germany). The results were expressed as micromoles of Trolox equivalents per gram of fresh weight (mg TE/g FW) using Trolox calibration curves (y = 0.0260x + 0.0816, R^2^ = 0.9916). All determinations were carried out in triplicate.

### 4.7. Total Monomeric Anthocyanin (TMA)

TMA content was quantified using the pH differential method following AOAC Official Method 2005.02 [38]. Approximately 0.3 g of fresh blueberries was ground in a mortar and mixed with 5 mL of methanol acidified with 1% HCl. The mixture was shaken at 500 rpm for 15 min, centrifuged at 5000 rpm at 4 °C for 5 min, and the residue was re-extracted twice with 5 mL of the same solvent. The combined extracts were adjusted to 15 mL. Absorbance was measured at 530 nm and 700 nm in buffers of pH 1.0 and pH 4.5, using a spectrophotometer (Specord 210 Plus, Analytik Jena, Jena, Germany). Results were expressed as cyanidin-3-*O*-glucoside equivalents per 100 g of fresh weight (mg CGE/100 g FW) using an extinction coefficient of 34,300 L cm^−1^ mol^−1^ [39].

### 4.8. Analysis of Anthocyanins by UPLC-PDA

#### 4.8.1. Extraction of Anthocyanins

The extraction procedure was performed following the method described by Lätti et al. [32] with minor modifications. Freeze-dried blueberries were ground into a fine powder, and 0.3 g of the sample was weighed into a centrifuge tube. Then, 5 mL of extraction solution was added. The extraction solvent consisted of 10% of acetonitrile–methanol (ACN–MeOH, 85:15 *v*/*v*) and 90% of 8.5% aqueous formic acid (HCOOH). The mixture was vortexed for 1 min, sonicated for 10 min, vortexed again, and centrifuged at 4500 rpm for 5 min at 4 °C. The residue was re-extracted three times. The supernatants were combined, and the final volume was adjusted to 15 mL. Prior to HPLC analysis, all extracts were filtered through a 0.13 µm syringe filter of regenerated cellulose.

#### 4.8.2. Qualitative and Quantitative Analyses of Anthocyanins

Separation and identification of anthocyanins were performed using a Waters ACQUITY UPLC System (Milford, MA, USA) coupled to UV/VIS diode-array detector (UV/VIS PDA) [32]. Separation was performed on a reverse-phase Zorbax Eclipse Plus C18 column (100 mm × 2.1 mm i.d., 1.8 µm; Agilent Technologies, Santa Clara, CA, USA) at 30 °C. A binary solvent system with solvent A (8.5% formic acid in water, *v*/*v*) and solvent B (ACN–MeOH, 85:15 *v*/*v*) and the following elution gradient was used: 0–3 min, linear 5–10% B; 3–5 min, linear 10–20% B; 5–7 min, linear 20–25% B; 7–8 min, linear 25–28% B; 8–9 min, linear 28–30% B; 9–10 min, linear 30–35% B; 10–18 min, linear 35–80% B; 18–20 min, isocratic 80% B; 20–23 min, linear 80–20% B; 23–25 min, linear 20–5% B; 25–30 min, isocratic 5% B. The volume of extract injected was 2 μL at a flow rate of 0.15 mL/min. The detection was recorded at 520 nm. Identification was based on retention time, UV spectra, comparison with commercial standards, and the literature [18,32].

Quantification was carried out using an external calibration curve (R^2^ ≥ 0.995) prepared from the pure standard of cyanidin-3-*O*-glucoside at known concentrations (1.0, 5.0, 10.0, 20.0, 35.0 and 50.0 µg/mL). The results were expressed as milligrams per 100 g of fresh weight (mg/100 g FW). All samples were injected in triplicate from independently prepared solutions of extracts.

### 4.9. Climatic Conditions

Climatic data on temperatures, sunshine duration, and level of precipitation recorded during the fruit development and ripening periods of the three harvest seasons (2023, 2024, and 2025) were obtained from the Pitești-Mărăcineni weather station and are presented in Table S1 (Supplementary Materials). These data were used to contextualize the observed inter-annual variation in fruit quality and phytochemical parameters in relation to the prevailing climatic conditions during each growing season.

### 4.10. Statistical Analysis

Statistical analysis was performed using two-way analysis of variance (two-way ANOVA, Genotype × Year) to assess the effects of genotype, harvest year, and their interaction on all physicochemical parameters. The analysis was conducted using the General Linear Model (GLM) Univariate procedure with Type III Sums of Squares in SPSS v27 (IBM Corp., Armonk, NY, USA), which is appropriate for the unbalanced design resulting from the absence of ‘Safir’ data in 2023. Post hoc comparisons were performed using Tukey’s HSD test at a 95% confidence level. Effect sizes were expressed as partial eta squared (η^2^p). For phenolic parameters (TPC, TMA, antioxidant activity, and individual anthocyanins), for which data were available for only two years (2024 and 2025), statistical analysis was performed using one-way ANOVA to identify significant differences, with post hoc comparisons conducted using the Tukey–Kramer test at a 95% confidence level. The analysis was carried out using XLStat software (version 2025.2.0 (1423), Addinsoft, New York, NY, USA). Data are expressed as mean ± standard deviation (SD).

## 5. Conclusions

The present study provides a comprehensive evaluation of fruit quality, phenolic content, anthocyanin profile, and antioxidant activity in the nine Romanian blueberry (*Vaccinium corymbosum* L.) genotypes over three consecutive harvest seasons. The investigated blueberry genotypes exhibited significant variability in fruit quality and phytochemical composition across years, highlighting strong genotypic effects. ‘Augusta’ and ‘Azur’ combined superior fruit size with favourable soluble solids-to-acidity balance, whereas ‘Prod’ showed consistently high firmness, supporting its suitability for postharvest handling. In contrast, ‘Delicia’ was characterized by higher acidity. No relationship was observed between soluble solids and acidity, indicating independent metabolic regulation.

Marked differences were also found in phytochemical profiles. Total phenolics and anthocyanins varied widely among genotypes, with ‘Lax’ and ‘Simultan’ showing the highest anthocyanin content and antioxidant capacity, while ‘Prod’ consistently exhibited the lowest levels. Qualitative analysis enabled the accurate identification of the ten anthocyanins belonging to the five major anthocyanidin classes, predominantly as glycosylated forms typical of *Vaccinium corymbosum* fruit.

The results demonstrate that both genotype and harvest year influenced the investigated characteristics, confirming that fruit phenotype in blueberries is determined by the interaction between genetic background and environmental conditions.

These findings highlight complementary functional traits among Romanian genotypes, supporting their targeted use in fresh consumption, nutraceutical applications, and value-added products enriched in natural antioxidants and anthocyanins.

## Figures and Tables

**Table 1:** Total phenolic content, total monomeric anthocyanin, antioxidant activity and anthocyanin contents in blueberry fruit genotypes for three different years.

Blueberry Genotypes/ Harvest Year	Total Phenolic Content (mg GAE/100 g FW)	Total Monomeric Anthocyanin (mg CGE/100 g FW)	Antioxidant Activity (mg TE/100 g FW)	Sum of Anthocyanin Compounds (mg/100 g FW)
**Augusta**				
2023	-	-	-	188.07 ± 2.70 ^d′^
2024	269.88 ± 19.65 ^B,C^	208.71 ± 14.49 ^B^	3471.63 ± 102.94 ^B,C,D^	475.57 ± 16.81 ^B^
2025	441.19 ± 67.34 ^a^	155.65 ± 25.31 ^d,e^	3503.50 ± 119.76 ^c,d^	388.30 ± 5.90 ^c,d^
**Azur**				
2023	-	-	-	219.49 ± 13.80 ^d′^
2024	258.28 ± 50.36 ^B,C^	162.25 ± 9.50 ^B,C^	3088.51 ± 70.90 ^C,D^	372.01 ± 19.66 ^C,D^
2025	307.10 ± 14.15 ^b,c^	179.42 ± 19.39 ^c,d^	3078.47 ± 181.99 ^d,e^	317.58 ± 22.95 ^d^
**Delicia**				
2023	-	-	-	453.47 ± 6.26 ^b′^
2024	292.86 ± 2.56 ^A,B^	208.73 ± 2.54 ^B^	3760.47 ± 82.06 ^B,C^	479.64 ± 14.43 ^B^
2025	287.53 ± 18.08 ^c^	127.27 ± 16.85 ^e,f^	3241.39 ± 174.27 ^d,e^	396.75 ± 30.26 ^c,d^
**Lax**				
2023	-	-	-	678.34 ± 28.63 ^a′^
2024	278.99 ± 39.10 ^A,B^	215.32 ± 29.11 ^B^	3526.06 ± 375.24 ^B,C^	629.37 ± 37.35 _A_
2025	385.88 ± 16.46 ^a,b^	312.77 ± 5.29 ^a^	3985.62 ± 342.71 ^a,b,c^	596.86 ± 36.99 ^a^
**Pastel**				
2023	-	-	-	420.51 ± 57.53 ^b′^
2024	299.19 ± 16.13 ^A,B^	183.42 ± 7.82 ^B,C^	3522.49 ± 222.36 ^B,C,D^	497.74 ± 39.42 ^B^
2025	324.79 ± 19.42 ^b,c^	159.86 ± 23.11 ^d,e^	3757.57 ± 249.59 ^a,b,c,d^	492.78 ± 42.07 ^b^
**Prod**				
2023	-	-	-	293.36 ± 18.08 ^c′^
2024	200.17 ± 10.74 ^C^	98.24 ± 18.46 ^D^	2736.98 ± 144.32 ^D^	298.29 ± 6.10 ^D^
2025	183.82 ± 8.80 ^d^	103.81 ± 8.21 ^f^	2698.56 ± 163.54 ^e^	300.24 ± 30.80 ^d^
**Safir**				
2023	-	-	-	-
2024	353.06 ± 25.57 ^A^	147.52 ± 22.66 ^C,D^	4204.11 ± 252.59 ^A,B^	482.33 ± 22.57 ^B^
2025	363.77 ± 36.32 ^a,b,c^	209.91 ± 3.58 ^c^	4312.07 ± 243.73 ^a,b^	428.25 ± 36.86 ^b,c^
**Simultan**				
2023	-	-	-	341.79 ± 22.77 ^c′^
2024	311.98 ± 30.83 ^A,B^	292.39 ± 6.31 ^A^	4709.42 ± 493.22 ^A^	520.99 ± 27.83 ^B^
2025	337.67 ± 50.33 ^b,c^	265.50 ± 20.84 ^b^	4470.53 ± 471.01 ^a^	440.79 ± 36.00 ^b,c^
**Vital**				
2023	-	-	-	310.60 ± 6.22 ^c′^
2024	289.28 ± 8.83 ^A,B^	141.29 ± 12.82 ^C,D^	3659.96 ± 108.71 ^B,C^	398.83 ± 20.55 ^C^
2025	302.10 ± 3.10 ^b,c^	144.77 ± 8.83 ^d,e,f^	3646.01 ± 123.78 ^b,c,d^	394.20 ± 47.29 ^c,d^

Values represented mean ± SD (*n* = 3). FW: fresh matter. - means not determined. Different letters indicate significant differences at *p* < 0.05 according to one-way ANOVA followed by Tukey’s HSD test: small letters with a prime are used to compare the samples from 2023, capital letters are used to compare the samples from 2024, small letters are used for those from 2025.

**Table 2:** Individual anthocyanin contents in blueberry fruit genotypes for three different years.

Genotypes/ Harvest Year	Dp-3-*O*-Gal	Dp-3-*O*-Glc	Cy-3-*O*-Gal	Cy-3-*O*-Glc	Pt-3-*O*-Glc + Cy-3-*O*-Ara	Pn-3-*O*-Gal	Pn-3-O-Glc + Mv-3-O-Gal	Mv-3-O-Glc
**Pick No.**	**1**	**2**	**3**	**4**	**5,6**	**7**	**8,9**	**10**
**Augusta**								
2023	19.89 ± 0.46 ^e′^	nd	18.33 ± 0.38 ^e′^	nd	13.05 ± 0.02 ^f′^	12.02 ± 0.28 ^c′^	16.80 ± 0.33 ^e′^	13.02 ± 0.00 ^d′^
2024	38.76 ± 1.64 ^C,D^	18.58 ± 0.39 ^E^	33.38 ± 1.35 ^C,D^	12.79 ± 0.05 ^D^	18.90 ± 0.35 ^D^	18.15 ± 0.62 ^B,C^	44.08 ± 1.98 ^C^	39.31 ± 2.28 ^C^
2025	26.56 ± 1.17 ^d^	15.40 ± 0.24 ^d^	23.24 ± 0.67 ^b^	12.84 ± 0.04 ^a^	16.48 ± 0.26 ^c,d^	14.50 ± 0.42 ^b^	28.42 ± 1.32 ^a,b^	26.69 ± 1.15 ^c^
**Azur**								
2023	27.15 ± 3.19 ^e′,f′^	nd	22.78 ± 2.04 ^d′,e′^	nd	13.52 ± 0.10 ^f′^	14.00 ± 0.89 ^c′^	20.15 ± 1.37 ^d′,e′^	nd
2024	60.88 ± 3.85 ^B^	12.32 ± 0.03 ^F^	39.35 ± 2.21 ^B,C^	nd	14.17 ± 0.15 ^E^	20.57 ± 0.68 ^B^	68.65 ± 5.79 ^A^	nd
2025	33.73 ± 2.47 ^c,d^	12.70 ± 0.01 ^d^	25.42 ± 1.30 ^b^	nd	14.37 ± 0.13 ^d^	14.82 ± 0.77 ^b^	23.86 ± 1.80 ^b^	nd
**Delicia**								
2023	45.01 ± 1.01 ^b′,c′^	31.28 ± 0.61 ^a′,b′^	32.16 ± 0.45 ^b′,c′^	15.83 ± 0.07 ^a′^	25.57 ± 0.30 ^b′^	14.09 ± 0.09 ^c′^	25.60 ± 0.36 ^b′,c′,d′^	38.62 ± 1.22 ^b′^
2024	34.52 ± 1.30 ^C,D^	26.99 ± 0.94 ^C,D^	27.42 ± 0.91 ^D^	15.00 ± 0.12 ^B,C^	24.48 ± 0.51 ^B,C^	14.44 ± 0.23 ^D^	29.71 ± 0.58 ^D^	48.77 ± 3.00 ^B,C^
2025	24.28 ± 2.16 ^d^	19.17 ± 1.22 ^c,d^	20.23 ± 1.26 ^b^	13.59 ± 0.23 ^a^	18.39 ± 1.07 ^c,d^	12.48 ± 0.68 ^b^	21.22 ± 2.03 ^b^	28.23 ± 3.25 ^c^
**Lax**								
2023	93.60 ± 4.65 ^a′^	36.32 ± 1.56 ^a′^	70.36 ± 3.30 ^a′^	15.04 ± 0.15 ^b′^	33.33 ± 1.15 ^a′^	27.75 ± 1.23 ^a′^	54.53 ± 2.55 ^a′^	69.47 ± 4.23 ^a′^
2024	85.11 ± 3.87 ^A^	33.96 ± 1.33 ^A^	69.57 ± 5.01 ^A^	16.94 ± 1.20 ^A^	34.57 ± 3.42 ^A^	27.48 ± 2.41 ^C^	58.61 ± 3.54 ^B^	80.90 ± 5.19 ^A^
2025	63.90 ± 4.32 ^a^	32.06 ± 3.39 ^a^	49.77 ± 4.41 ^a^	15.57 ± 0.39 ^a^	31.05 ± 3.96 ^a^	22.25 ± 3.16 ^a^	36.73 ± 4.14 ^a^	52.87 ± 7.29 ^a,b^
**Pastel**								
2023	51.61 ± 11.41 ^b′^	27.16 ± 4.56 ^b′^	34.56 ± 6.62 ^b′^	13.03 ± 0.19 ^c′^	23.38 ± 3.08 ^b′,c′^	16.87 ± 2.27 ^b′^	26.66 ± 4.06 ^b′,c′^	35.75 ± 6.84 ^b′^
2024	54.17 ± 5.71 ^B^	32.27 ± 2.85 ^A,B^	37.52 ± 3.60 ^B,C^	13.90 ± 0.24 ^C,D^	28.24 ± 2.18 ^B^	17.89 ± 1.24 ^B,C,D^	36.34 ± 3.27 ^C,D^	61.39 ± 6.43 ^B^
2025	47.47 ± 4.97 ^b^	31.01 ± 2.50 ^a^	31.94 ± 2.83 ^b^	14.12 ± 0.14 ^a^	25.71 ± 1.41 ^b^	16.52 ± 0.70 ^b^	28.97 ± 1.83 ^a,b^	48.96 ± 3.90 ^b^
**Prod**								
2023	40.56 ± 3.62 ^c′,d′,e′^	nd	29.74 ± 2.01 ^b′,c′,d′^	nd	13.84 ± 0.11 ^e′,f′^	17.59 ± 1.07 ^b′^	31.70 ± 2.45 ^b′^	nd
2024	33.89 ± 0.83 ^C,D^	nd	27.43 ± 0.57 ^D^	nd	13.58 ± 0.05 ^E^	16.57 ± 0.31 ^C,D^	36.23 ± 1.03 ^C,D^	nd
2025	27.50 ± 2.88 ^d^	nd	23.03 ± 2.15 ^b^	nd	14.16 ± 0.51 ^d^	14.90 ± 0.98 ^b^	27.57 ± 3.07 ^b^	nd
**Safir**								
2023	nd	nd	nd	nd	nd	nd	nd	nd
2024	29.71 ± 1.48 ^D^	30.23 ± 1.65 ^A,B,C^	26.11 ± 1.18 ^D^	15.76 ± 0.28 ^A,B^	28.44 ± 1.44 ^B^	15.81 ± 0.60 ^C,D^	35.79 ± 2.02 ^C,D^	86.12 ± 6.32 ^A^
2025	25.56 ± 2.16 ^d^	28.16 ± 2.46 ^a,b^	22.58 ± 1.68 ^b^	15.12 ± 0.38 ^a^	24.84 ± 1.63 ^b^	14.27 ± 0.68 ^b^	26.03 ± 1.80 ^b^	64.11 ± 6.89 ^a^
**Simultan**								
2023	29.23 ± 3.59 ^d′,e′,f′^	17.94 ± 1.17 ^c′^	25.72 ± 2.80 ^c′,d′,e′^	12.92 ± 0.13 ^c′^	16.90 ± 0.79 ^d′^	13.12 ± 0.88 ^c′^	20.55 ± 1.81 ^d′,e′^	21.87 ± 2.04 ^c′,d′^
2024	53.10 ± 3.92 ^B^	28.24 ± 1.05 ^B,C,D^	45.29 ± 3.84 ^B^	13.95 ± 0.17 ^C,D^	23.63 ± 1.29 ^C^	18.31 ± 1.07 ^B,C^	35.96 ± 2.21 ^C,D^	44.14 ± 1.92 ^C^
2025	37.79 ± 5.34 ^b,c^	23.78 ± 2.55 ^b,c^	35.64 ± 5.74 ^b^	13.88 ± 0.28 ^a^	19.73 ± 1.47 ^c^	16.36 ± 2.30 ^b^	21.23 ± 1.88 ^b^	26.87 ± 2.72 ^c^
**Vital**								
2023	24.73 ± 0.90 ^f′^	16.18 ± 0.40 ^c′^	23.48 ± 0.75 ^d′,e′^	12.66 ± 0.04 ^c′^	16.63 ± 0.22 ^d′,e′^	14.03 ± 0.35 ^c′^	19.84 ± 0.51 ^d′,e′^	22.77 ± 0.71 ^c′^
2024	42.77 ± 2.26 ^C^	25.02 ± 1.05 ^D^	36.86 ± 2.04 ^C^	13.37 ± 0.08 ^D^	22.97 ± 0.79 ^C,D^	18.94 ± 0.83 ^B,C^	36.98 ± 1.97 ^C,D^	59.31 ± 4.06 ^B^
2025	25.07 ± 2.99 ^d^	17.34 ± 1.15 ^d^	22.25 ± 2.28 ^b^	13.04 ± 0.12 ^a^	17.62 ± 0.89 ^c,d^	13.53 ± 0.67 ^b^	22.30 ± 1.77 ^b^	31.79 ± 3.53 ^c^

Values represent mean (mg/100 g FW) ± SD (*n* = 3). Dp: delphinidin; Cy: cyanidin; Pt: petunidin; Pn: peonidin; Mv: malvidin; Gal: galactoside; Glc: glucoside; Ara: arabinoside. Compounds were identified by comparison with a standard. nd—not detected. Different letters indicate significant differences at *p* < 0.05 according to one-way ANOVA followed by Tukey’s HSD test: small letters with a prime are used to compare the samples from 2023, capital letters are used to compare the samples from 2024, small letters are used for those from 2025.

## Data Availability

The original contributions presented in this study are included in the article/Supplementary Material. Further inquiries can be directed to the corresponding author.

## Supplementary Materials

The following supporting information can be downloaded at https://www.mdpi.com/article/10.3390/molecules31122068/s1: Table S1: Monthly climatic data recorded at the Pitești-Mărăcineni weather station (287 m altitude; 44.89° N, 24.87° E) during the three harvest years (2023–2025).

## Abbreviations

The following abbreviations are used in this manuscript:

AA	Antioxidant activity
AFW	Average fruit weight
AOAC	Association of Official Analytical Chemists
CGE	Cyanidin-3-*O*-glucoside equivalents
DM	Dry matter content
DPPH	2,2-diphenyl-1-picrylhydrazyl
FW	Fresh weight
G × E	Genotype × environment interaction
GAE	Gallic acid equivalents
PDA	Photodiode array
SD	Standard deviation
SRAP	Sequence-related amplified polymorphism
TA	Titratable acidity
TE	Trolox equivalents
TMA	Total monomeric anthocyanin content
TPC	Total phenolic content
TSS	Total soluble solids
UPLC	Ultra-performance liquid chromatography
UV-Vis	Ultraviolet-visible

## Appendix A

**Table A1:** Two-Way ANOVA (Genotype × Year) results for physicochemical parameters of *V. corymbosum* Fruits (SPSS v27, GLM Univariate, Type III SS).

Parameter	Unit	Genotype F (df)	Genotype p	Genotype η^2^p	Year F (df)	Year p	Year η^2^p	Genotype × Year F (df)	Genotype × Year p	Genotype × Year η^2^p	R^2^ (adj. R^2^)	Levene’s p
Fruit mass	g	F_8,216_ = 26.210	<0.001	0.493	F_2,216_ = 427.677	<0.001	0.798	F_15,216_ = 8.838	<0.001	0.380	0.844 (0.826)	0.120
Polar dimensions	mm	F_8,234_ = 16.454	<0.001	0.360	F_2,234_ = 140.971	<0.001	0.546	F_15,234_ = 4.445	<0.001	0.222	0.660 (0.623)	0.529
Equatorial dimensions	mm	F_8,234_ = 40.457	<0.001	0.580	F_2,234_ = 239.026	<0.001	0.671	F_15,234_ = 4.221	<0.001	0.213	0.781 (0.757)	0.567
Firmness	N	F_8,198_ = 7.940	<0.001	0.243	F_2,198_ = 53.529	<0.001	0.351	F_15,198_ = 6.954	<0.001	0.345	0.566 (0.511)	0.589
Total soluble solids	% Brix	F_8,219_ = 10.132	<0.001	0.270	F_2,219_ = 16.750	<0.001	0.133	F_15,219_ = 5.561	<0.001	0.276	0.476 (0.416)	0.002 *
Titratable acidity	g malic ac./100 g FW	F_8,52_ = 880.323	<0.001	0.993	F_2,52_ = 115.160	<0.001	0.816	F_15,52_ = 71.885	<0.001	0.954	0.994 (0.991)	<0.001 *
Dry matter	%	F_8,50_ = 7.415	<0.001	0.543	F_2,50_ = 18.063	<0.001	0.419	F_15,50_ = 2.405	0.01	0.419	0.722 (0.583)	<0.001 *

Notes: df = degrees of freedom; η^2^p = partial eta squared; R^2^ = model fit (adjusted R^2^ in parentheses). * Levene’s test *p* < 0.05 indicates heterogeneity of error variances; ANOVA results are considered robust given the relatively balanced sample sizes. ‘Safir’ cultivar has no data for 2023 (unbalanced design); Type III Sums of Squares applied throughout.

**Table A2:** Dry matter content (%) in nine *V. corymbosum* genotypes across three harvest years (means ± SD; *n* = 2–3).

Cultivar	2023	2024	2025
Augusta	15.64 ± 0.57 abcd	13.29 ± 0.62 d	16.65 ± 1.72 abcd
Azur	18.65 ± 0.57 ab	16.46 ± 1.08 abcd	16.51 ± 1.58 abcd
Delicia	17.95 ± 0.28 abc	16.29 ± 0.57 abcd	15.8 ± 1.35 abcd
Lax	19.98 ± 5.35 a	14.26 ± 1.17 bcd	14.19 ± 1.14 bcd
Pastel	17.69 ± 2.04 abcd	15.09 ± 0.8 abcd	17.53 ± 0.62 abcd
Prod	13.5 ± 0.85 cd	14.0 ± 1.69 cd	14.68 ± 1.34 bcd
Safir	n.d.	15.41 ± 0.28 abcd	18.09 ± 0.5 abc
Simultan	19.58 ± 2.12 a	16.61 ± 0.95 abcd	19.9 ± 2.7 a
Vital	15.76 ± 1.16 abcd	13.68 ± 0.31 cd	17.35 ± 1.32 abcd

Different letters indicate significant differences at *p* < 0.05 according to two-way ANOVA (Genotype × Year) followed by Tukey’s HSD test (HSD.test, agricolae package, R Studio version 2026.01.2, Build 418; Posit Software, PBC, Boston, MA, USA). n.d.: not determined.
